# Hospital Meetings, &c.

**Published:** 1900-05-26

**Authors:** 


### HOSPITAL MEETINGS, &C.

### HAMPSTEAD HOSPITAL.

Biennial Festival.—A Record Total.

The biennial festival dinner in connection with the Hampstead Hospital was held at the Grand Hotel on Wednesday evening, May 16th, the Earl of Mansfield presiding over a large company.

The usual loyal toasts were honoured, the Chairman referring to the interest which the Prince and Princess of Wales take in hospitals; to "the glorious first of June last year, when the Princess Christian did a great deal for us at Golder's Hill;" and to the assistance of the Duchess of Albany, who, with other ladies of the Middlesex Needlework Guild, had provided clothes and other necessaries for the hospital.

Mr. E. Bond, M.P., gave "Our Imperial Forces"—an old toast, he remarked, under a new name, and he confessed he liked the new name in its comprehensiveness and its catholicity. Not least among these forces was the Army Medical Corps, which was rendering such effective and admirable service in South Africa. (Hear, hear.)

Major-General Henry Trotter (commanding the Home District), whose name was associated with the toast, offered a brief acknowledgment.

Mr. J. S. Fletcher, J.P., submitted "The Houses of Parliament," and, in coupling the names of Lord Mansfield and Mr. E. Brodie Hoare, M.P., with the toast, remarked upon the kindly interest his lordship took in Hampstead and its institutions and people.

The gentlemen named having replied,

The toast of the evening, "Prosperity to the Hampstead Hospital," was proposed by the Chairman in very felicitous terms. His lordship said he appeared before those present as an humble but importunate beggar, and he asked their kind consideration towards him in that capacity. He was pleading for a very deserving institution when he asked for support for the Hampstead Hospital. (Hear, hear.) It was obvious that an institution such as this hospital could not be allowed to stand still; it must be enlarged and, he supposed, removed somewhere else. All present doubtless knew of the schemes on foot—or he might, perhaps, say in the air—for the enlargement and possible removal of the hospital, and he trusted they would all do everything in their power to bring to a successful issue whatever scheme might ultimately be decided upon. (Applause.)

The toast was enthusiastically honoured, and Mr. W. F. Malcolm (the treasurer), in response, said that they threw themselves on the generosity of their friends in Hampstead, and when the secretary proclaimed the result he thought the confidence of the committee would be found to have been justified. It was at the dinner two years ago that the proposed new hospital, in substitution for the present somewhat makeshift one, came for the first time within the range of practical politics. They had to thank many kind friends in Hampstead for the manner in which they had responded to the appeal put forward for the construction of the new hospital, and also to thank H.R.H. Princess Christian very heartily not only for being a patron, but also for being personally present at the meeting on June 1st last year, to which the hospital owed such a large amount of support towards the construction of the new building. They had also to thank the Ladies' Association, without whose kind help they could not have made the progress they had done. There was one matter of which he was rather ashamed, and that was that they had not yet got a site for the new hospital. The difficulty of obtaining a suitable place—which, by the way, was evidence of the prosperity of Hampstead—had been very great indeed, but they hoped they would succeed ere long in securing one, and when they had done so he was sure the generosity already shown by the inhabitants of Hampstead would be renewed, and that they would be enabled to build a hospital which would be a credit to the place. (Applause.) When they had secured a site they would again become ardent beggars for the construction of the hospital. (Applause.)

Mr. B. A. Lyon proposed "The Council, Treasurer, and Hon. Secretary," to which Mr. J. McMillan replied.

Mr. R. A. Owthwaite, the hon. secretary, then announced amid loud cheers that the total proceeds of the festival amounted to £2,023 (including over £100 in new annual subscriptions), the Chairman's list being £1,171 17s. 6d. He added that this was a record total, the highest figure reached previously being about £1,600.

The other toasts were "The Medical Staff," proposed by Mr. Frank Debenham, and responded to by Mr. Edmund Owen and Dr. W. Heath Strange; "The Stewards," given by the Chairman, and replied to by Mr. H. W. Birks; and "The Chairman."

